# The Production of Carbon Nanofiber on Rubber Fruit Shell-Derived Activated Carbon by Chemical Activation and Hydrothermal Process with Low Temperature

**DOI:** 10.3390/nano11082038

**Published:** 2021-08-10

**Authors:** Suhdi Suhdi, Sheng-Chang Wang

**Affiliations:** Department of Mechanical Engineering, Southern Taiwan University of Science and Technology, Tainan City 71005, Taiwan; da61y203@stust.edu.tw

**Keywords:** rubber fruit shell, nanofibers, hydrothermal

## Abstract

Recently, the conversion of biomass into carbon nanofibers has been extensively studied. In this study, carbon nanofibers (CNFs) were prepared from rubber fruit shell (RFS) by chemical activation with H_3_PO_4_, followed by a simple hydrothermal process at low temperature and without a vacuum and gas catalyst. XRD and Raman studies show that the structure formed is an amorphous graphite formation. From the thermal analysis, it is shown that CNFs have a high thermal stability. Furthermore, an SEM/TEM analysis showed that CNFs’ morphology varied in size and thickness. The obtained results reveal that by converting RFS into an amorphous carbon through chemical activation and hydrothermal processes, RFS is considered a potential biomass source material to produce carbon nanofibers.

## 1. Introduction

As is known, carbon nanofibers (CNFs) are an essential material of carbon. That has been widely studied in various basic scientific research and industrial applications. CNFs are one of the most important members of carbon fibers that are usually useful in many applications due to their unique mechanical, physical, chemical, and electrical [1,2] properties, and they also have fibrous, cylindrical, and cup-stacked structures that can produce a nano-scaled quantum effect [3]. CNFs are carbon allotropes with a diameter of around 50–100 nm and a length of between one and a hundred micrometers [4].

The utilization of carbon nanofibers (CNFs) as a potential material has recently been demonstrated in a variety of fields, such as adsorbents [5,6], sensors [2,7], electrode materials and electromagnetic shielding [8], batteries, solar cells [9], supercapacitors [3], gas storage [10,11], and biomedical applications [12,13,14]. Due to the rising usage of CNFs in various applications, the demand for CNF has risen in recent years, accompanied by an increase in selling prices. As a result, alternate approaches to producing low-cost CNFs must be identified. In most cases, these initiatives aim to lower costs by using less expensive materials, lowering costs through processing or combining the two approaches.

Numerous kinds of CNF have been produced recently from a variety of sources. Nevertheless, the majority of CNFs are derived from non-renewable precursors [15]. Due to their limitations and unsustainable characteristics as significant concerns, the organic polymer sources from biomass have been heavily studied in recent years, such as bamboo [16], mango seed and bleached Kraft pulp [6], sawdust [17], crab shell [18], and natural fungus [19]. Biomass is a lignocellulosic plant material composed of different polymers, including cellulose (40–50%), hemicellulose (20–40%), and lignin (10–15%) [20]. Lignin is a natural polymer that is the most effective replacement for producing carbon nanofibers, particularly with the addition of phosphoric acid (H_3_PO_4_) to the lignin solution, more curled fibers will form [5] and cellulose as cellulose nanofibers [21,22]. Exploring different resources is substantial considering that the characteristics of cellulosic material can be changed by plant age and location, seasonal climate conditions, and impurity extraction processes [23]. As a result, finding a bio-based precursor material for CNF synthesis is essential.

Rubber fruit is a potentially renewable resource for the production of CNF precursors since the shell of the rubber fruit (Hevea brasiliensis) is reported to contain between 35% and 54% lignin [6]. However, relatively few investigations/reports have been published on rubber fruit shells, considered potential precursors for carbon fiber synthesis. According to recent research, several studies have used rubber fruit as a raw material for various purposes. Some studies demonstrate the use of seeds as food, feed, and biofuel [24,25]. Several studies have utilized rubber seed shell (RSS) to produce high-quality activated carbon via physical activation with steam [26], a promising precursor of activated carbon for phenol removal from water [27], activated carbon as a potential CO2 removal, where the activation process is performed with malic acid [28,29] and the use of RSS as a filler in polymer technology [30]. While several studies employ rubber fruit shell (RFS) as a raw material, they include the production of activated carbon as a bio sorbent for removing free fatty acid (FFA) from crude palm oil (CPO) [6], producing activated carbon for wastewater treatment [31] and producing activated carbon as a pack carburizing medium in the heat treatment process [32]. From the information above, it is clear that RSS and RFS have only been used as precursors in the manufacture of activated carbon, but not yet in the manufacture of CNFs.

Indonesia is the second-largest producer of rubber in the world [33]. According to statistical data, Indonesia has the world’s largest rubber plantation area, at approximately 3,671,387 ha in 2020, with production reaching 3.54 million tons of rubber [34]. Rubber plantations have historically focused exclusively on latex and stem processing, with little attention paid to other products such as rubber fruit. Rubber fruit is a by-product of Indonesia’s extensive rubber plantations. Rubber fruit has historically had little economic value and has been used exclusively as a generative seed for rubber trees. The rubber fruit comprises three major components: the fruit shell, the seed shell, and the seed itself, with the fruit shell accounting for 70% of the total weight, 9% of the seed shell, and 21% of the seed [35]. Rubber plantations produce approximately 1000 kg of rubber fruit per hectare per year [4], equating to approximately 3.6 million tons of rubber fruit per year. Therefore, raw materials will be available in the form of rubber fruit shells (RFS) of 2.569 million tons per year, rubber seed shells (RSS) of 0.330 million tons per year, and rubber seeds (RS) of 0.771 million tons per year. This number is the large quantity of raw material as a greener precursor material derived from renewable plant-based resources, particularly for RFS, which is desirable for CNFs precursor synthesis.

Carbon nanofibers synthesized from biomass can be produced in various methods. The first is the electrospinning method [3,8,36], which consists of an electrospinning process followed by a heat treatment procedure, and it can produce multifunctional carbon materials such as carbon nanofibers (CNFs) with diameters ranging from a few tens of nanometers to several micrometers [21,22]. The biomass material is chemically treated to yield biomass fibers, which are then mixed with a polymer solution, placed in a capillary tube, and carbonized at temperature > 600 °C [3,22,37]. The second method is pyrolysis [23] which can also be used to produce crosslinked carbon nanofibers. During pyrolysis, the biomass formed by complex organic structures undergoes a series of decomposition steps, starting with the release of water vapor followed by the decomposition of hemicellulose, cellulose, and lignin. However, pyrolysis involves high electricity consumption because it needs to be carried out at high temperatures of up to 900 °C. The third method is ultrasonication [38] which degrades the material by using ultrasonic waves at a frequency higher than 20 kHz combined with a liquid medium such as a solvent or polymer melt [5]. The last and most frequently utilized method is hydrothermal synthesis [39,40,41]. The production of nanomaterials in hydrothermal synthesis may occur across a broad temperature range, from a low to extremely high temperature, and in either a low or high-pressure environment depending on the main composition [40]. Recently, many researchers have used hydrothermal synthesis at low temperatures because of its energy-saving property and simple synthesis. Manafi et al. [42] used an easy sonochemical/hydrothermal method at 160 °C. Funke et al. [43] used elevated temperatures (180–220 °C) for biomass in a water suspension under saturated pressure for a few hours. Deng et al. [19] extracted nanofibers from biomass carbon, Teflon-coated stainless steel autoclave was added, and 70 mL of 3 M KOH solution was added and treated at 150 °C. However, almost all of these methods are synthesized using catalysts such as Ni, Co, and Fe, and require gases and vacuum support to obtain nanostructured materials [23,44,45].

As mentioned above, several studies have demonstrated that precious CNFs may be produced from biomass feedstocks. However, the majority of these procedures require high reaction temperatures and metal catalysts. As a result, we used a more environmentally friendly precursor material obtained from a renewable plant resource (rubber fruit shell) to synthesize carbon nanofibers in the present study. The RFS as a precursor was carbonized via pyrolysis and then chemically activated with phosphoric acid (H_3_PO_4_), and then to obtain CNFs, the rubber fruit shell activated carbon (RFSAC) was carried out by a simple hydrothermal process without the addition of catalysts, at low temperature (90 °C), and vacuum and gasses were not required.

## 2. Materials and Methods

### 2.1. Material Preparation

**Figure 1:**
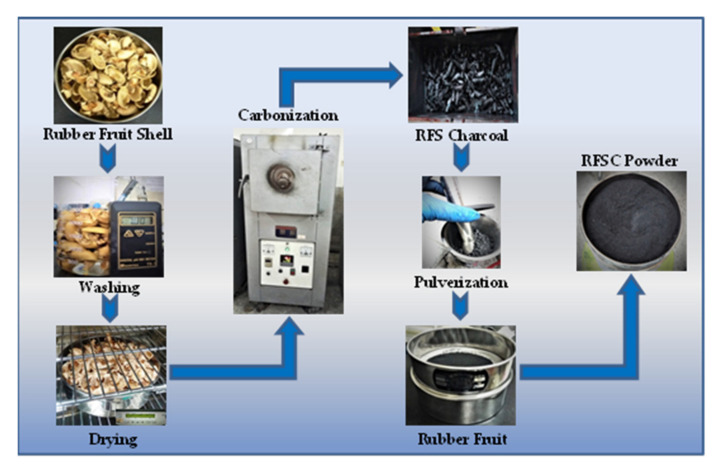
The rubber fruit shell charcoal (RFSC) powder manufacturing process flow.

In this study, the precursor was prepared from rubber fruit shells collected from Bangka Island of Indonesia through washing, drying, carbonization, pulverization, and dan sieving processes according to a previous study by the authors [32]. The RFS was first submerged in 10% H_2_SO_4_ for 4 h, rinsed in distilled water to remove soil contaminants, and dried at 60 °C for 24 h. The dried precursor was carbonized for 1 h at 450 °C in nitrogen (20 mL/min). Finally, The RFS were then crushed and sieved at 200 mesh (74 µm) sieve size as shown in Figure 1.

**Figure 2:**
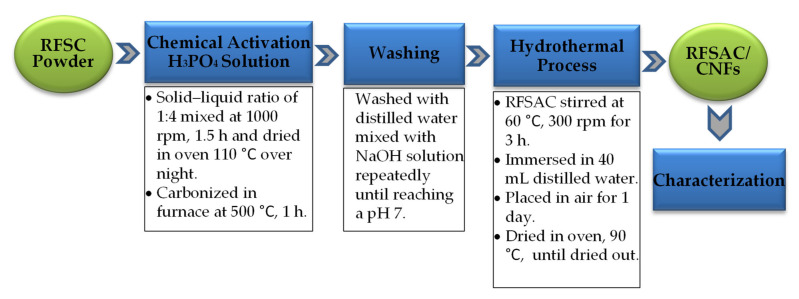
The chemical activation and hydrothermal processes.

The 3 g powder of rubber fruit shell charcoal (RFSC) was impregnated with phosphoric acid (H_3_PO_4_) 85% by weight solution in water supplied by Fisher Scientific [46,47]. The impregnation ratio between charcoal weight and the activating agent was 1:4 wt ratio. The slurry was mixed for 1.5 h at 300 rpm with a magnetic stirrer (Corning PC-420D, Corning Inc., Corning, NY, USA). The homogeneous slurry was dried in the oven (VENTICELL 55, MMM Medcenter Einrichtungen GmbH, Munich, Germany) at 110 °C overnight. Then, the black and sticky slurries were put in a ceramic crucible and carbonized in the furnace (Muffle furnace KL 03/11, THERMCONCEPT GmbH, Bremen, Germany) at 500 °C at a rate of 10 °C/min [46,47]. The activation time was in range of 60 min, in a self-generated atmosphere and cooled to room temperature. The samples of rubber fruit shell activated carbon (RFSAC) were washed using distilled water mixed with NaOH solution and centrifuged for 30 min at 1000 rpm repeatedly to remove the excess activating until a pH of around seven was achieved. After this process was complete, the hydrothermal process was performed. The supernatant was immersed in 40 mL distilled water and stirred at 60 °C, 300 rpm for 3 h, and was then placed in air for one day. In the last process, the solution was dried in the dry oven at 90 °C for around three days until complete dryness. The processes as shown in Figure 2.

### 2.2. Characterization

The thermal decomposition behavior of samples was analyzed by the Thermal Gravimetric Analysis (TGA) machine (METTLER TOLEDO-TGA/DSC1 STAR e System, Schweiz GmbH, Greifensee, Switzerland) was used. The analysis was performed by heating the 9 to 10 mg sample to 900 °C at a heating rate of 10 °C in a nitrogen atmosphere and a 50 mL/min flow rate. The curves of weight loss were re-plotted by Origin 8 software (OriginLab Corporation, Northampton, UK). The number of specific surface areas and pore volumes of sample products was determined by accelerated surface area and porosimetry system machine (ASAP 2020, Micromeritics Japan, G.K., Chiba, Japan). For this analysis, the sample’s 0.2–0.3 g was used with an automated degas cycle, N_2_ adsorptive analysis, −196 °C bath temperature analysis, 10 s equilibration time, an ambient temperature at 22 °C. The specific surface areas were recorded using the Brunauer–Emmett–Teller (BET) technique and the assessment of pore volumes was obtained using the Barrett–Joyner–Halenda (BJH) algorithm.

X-ray diffraction (Bruker, D2 Phaser, Karlsruhe, Germany) with Cu Kα radiation from 10° to 50° was used to characterize the phase and crystal structure of as-synthesized electrocatalysts. Raman spectroscopy analysis was performed using Laser Spectroscopy Technologies Confocal Micro and Nano Raman (UniDRON, CL Technology Co., Ltd., New Taipei, Taiwan). To find out the morphological structure and the composition of sample elements, the instrument used was Scanning Electron Microscope-Energy Dispersive Spectroscopy (JEOL JSM-6701F, JEOL Ltd., Tokyo, Japan) and High-resolution Transmission Electron Microscopy (JEOL-JEM-1230, JEOL Ltd., Tokyo, Japan). The size and number of CNFs formed were determined, the SEM images were characterized using ImageJ software version 2.1.0/53k, Java 1.8.0_172(64 bit). The samples were processed using conventional methods, while the number of samples was around 273 grains. Then, plotted using histogram diagrams and Gaussian curves in Origin 8.

## 3. Results

### 3.1. Thermogravimetric Analisys of RFSAC and Nanofibers

**Figure 3:**
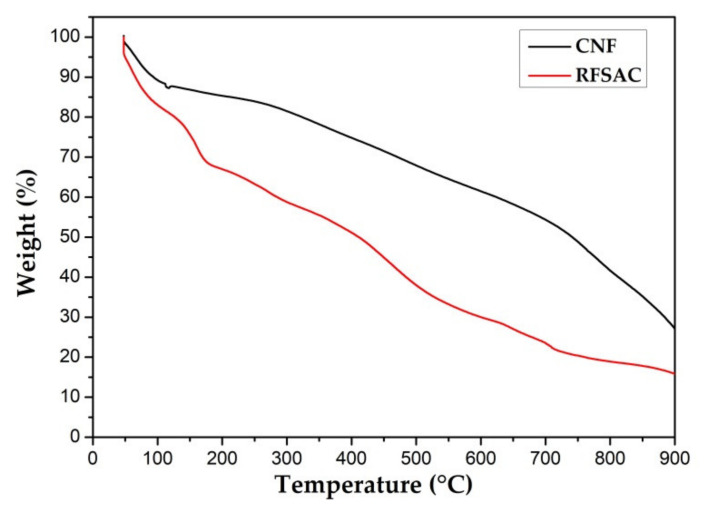
Thermal decomposition analysis result of RFSAC and carbon nanofibers.

Figure 3 shows the thermal decomposition behavior of the sample that was identified by thermogravimetric analysis (TGA). The curve shows that the weight loss of RFSAC started from 60 °C to 200 °C, and the significant mass loss occurred due to water dehydration on the surface. The RFSAC began to decompose at about 200 °C, reducing mass to around 80% at 700 °C; both the dehydrated water discharge and oxidation processes were responsible for the mass loss. The mass began to decrease steadily at higher temperatures (e.g., 700 to 900 °C) due to the release of carbon dioxide [48]. In comparison, carbon nanofibers began to decompose at temperatures 300 °C higher than RFSAC. At 900 °C, the residue left weight percentage of carbon nanofibers was 26.7% higher than that of the RFSAC (15.7%). The above results show that carbon nanofibers have a higher thermal stability.

### 3.2. BET Specific Surface Area and Porosity of RFSAC and Carbon Nanofibers

**Figure 4:**
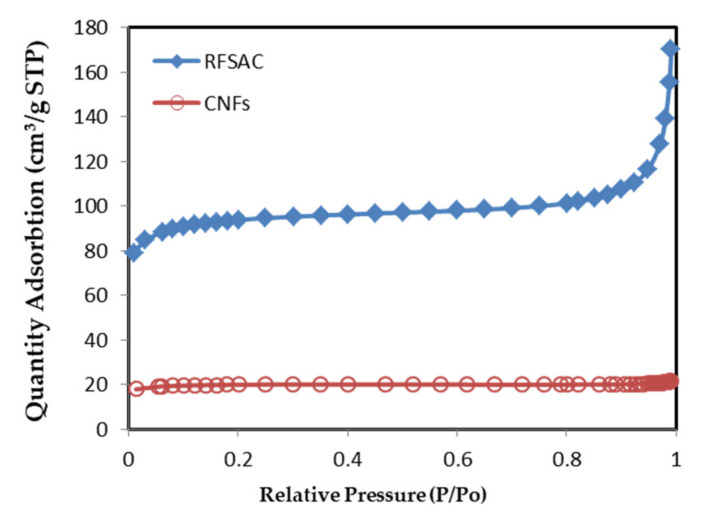
Nitrogen absorption isotherm RFSAC and carbon nanofibers.

The specific surface area and total pore volume (Vtotal, cm^2^/g) were measured by the BET method in the isotherm region and calculated by N_2_ gas adsorption on the surface (see Figure 4). The specific surface area of CNFs was 63 m^2^g^−1^ at P/P_0_ = 0.9902, while 286 m^2^g^−1^ for RFSAC at P/P_0_ = 0.9911. The adsorption average pore size of CNFs (2.11 nm) was lower than the RFSAC (2.76461 nm). Whereas in this case, the average pore size influenced the specific surface area values because the CNFs primarily retained their pore structure, their surface area may be lower than that of RFSAC, which was exfoliated. The decrease in the specific surface area from 286 to 63 m^2^g^−1^ was probably caused by the growth of CNFs that occurred on the surface or RFSAC pores, so that CNF not only moved to the RFSAC surface, but also lodged in the RFSAC pores. Several researchers observed similar findings, where they declared that CNFs/CNTs were found on the surface and within the pores of biomass-activated carbon-derived, such as; Su et al. [49] showed that following CNF formation, the specific surface area of palm shell-based AC was decreased from 1490 to 305 m^2^g^−1^. Similarly, Chen et al. [45] found that the specific surface areas of wheat straw and coconut-derived AC decreased substantially following CNT development, from 1121 to 102 m^2^g^−1^ and 1189 to 32 m^2^g^−1^, respectively.

### 3.3. The Element Content of Carbon Nanofibers Grown on RFSAC

**Figure 5:**
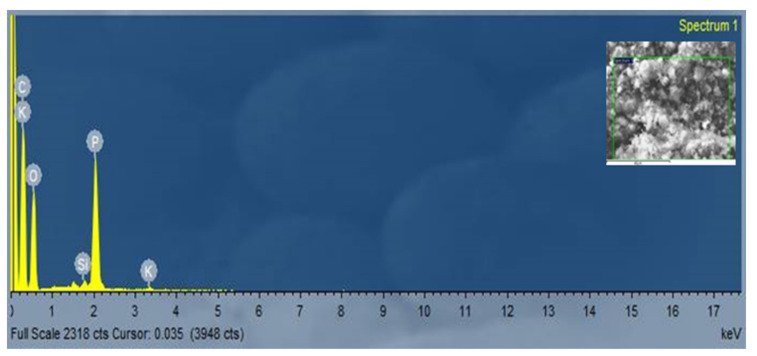
EDS spectrum of CNTs grown on the surface of RFSAC.

The element content of CNFs grown on the RFSAC surface was identified by EDS (see Figure 5), and the results are shown in Table 1. The three dominant elements produced were 62.48 at.% carbon, 32.83 at.% oxygen, and 4.38 at.% phosphorus, while other elements such as silicon and potassium were less than 1 at.%.

### 3.4. Morphology and Microstructure of CNTs

**Figure 6:**
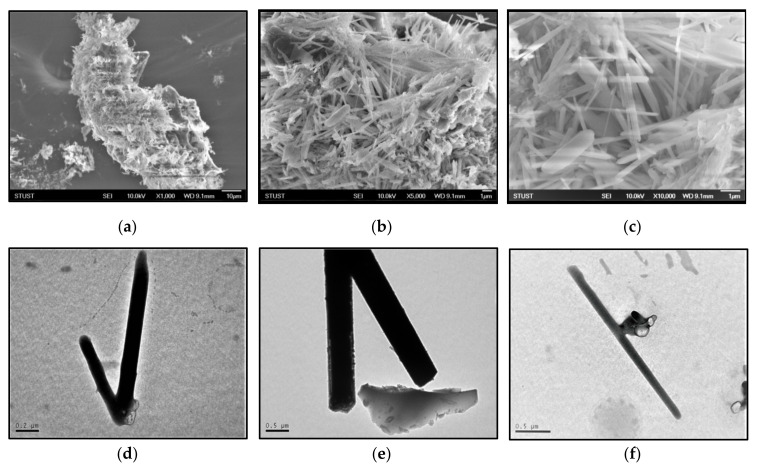
(**a**–**c**) SEM images and (**d**–**f**) TEM images of carbon nanofibers.

Figure 6a–c SEM images show the clusters of CNFs formed on the activated carbon surfaces. According to SEM analysis, enmeshed CNFs with varied diameters plentifully surrounded the surface of the RFSAC. The structure and dimensions of CNFs were more clearly shown in TEM images in Figure 6d–f, where the inhomogeneous dimensions of CNFs are visible. It was proven that this synthesis technique makes it possible to produce carbon nanofiber materials with varying diameters and lengths.

**Figure 7:**
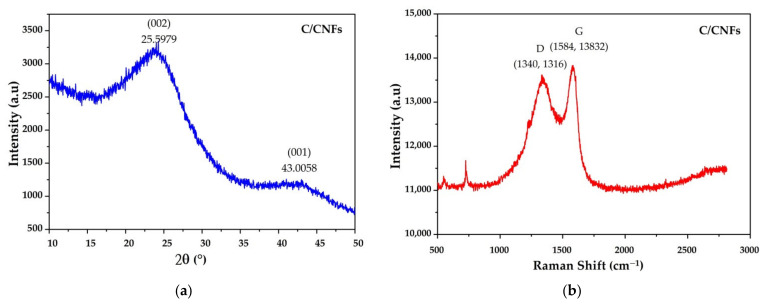
(**a**) The XRD pattern of CNFs and (**b**) Raman Spectra of CNFs.

The microstructure of CNFs was investigated by XRD and the respective patterns are shown in Figure 7a. In the result of XRD, there were two broad peaks 2θ at around 25.6°, and 43.0° can be indexed to the (002) and (001) diffraction plane of CNFs with an amorphous or low crystallinity carbon phase, and those peaks were characteristic crystal planes of graphitic carbon [50]. The Raman spectrum of the CNF is shown in Figure 7b; there were two prominent peaks positioned at around 1340 and 1582 cm^−1^ which were labeled as D and G bands of carbon atom crystal, respectively. The D band was associated with defects and disorders in carbon materials, whereas the G band reflected the vibrational mode of two carbon atoms moving in opposite directions in a graphite [51]. The intensity ratio (I_D_/I_G_) of the D and G bands represented the degree of the structural defect of the material and graphitization of the carbon nanofibers [50,51]. According to the Raman spectra, the I_D_/I_G_ of CNFs grown on the RFSAC’s surface was 0.98. A low intensity ratio value of I_D_/I_G_ indicated a dominant ordered structure or indicated a low level of structural defects in CNFs. On the other hand, if the value of the I_D_/I_G_ ratio was high, more structural defects were introduced to the carbon material.

**Figure 8:**
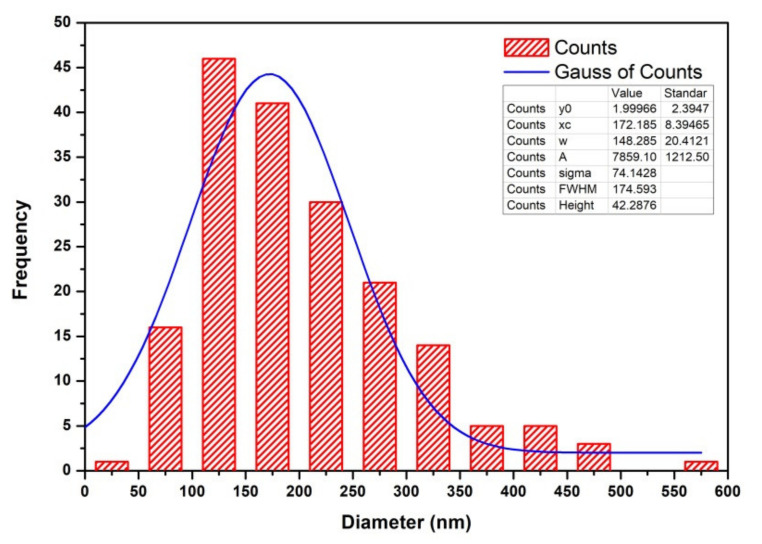
The histogram curve of CNFs diameter distribution.

ImageJ was used to obtain the data of CNFs’ size. Then, to obtain the CNFs’ diameter size distribution, the data were plotted in a histogram diagram analyzed using Origin 8. Figure 8 shows a histogram and Gaussian curve diagram of the CNFs’ size distribution, where the resulting curve was asymmetrical. It indicated that the diameter size distribution of CNFs grown on the RFS-activated carbon surface was not uniform or non-homogeneous. The diameter range was 38 nm and 554 nm. The most commonly formed diameter of CNFs was between 100 and 150 nm, and the average diameter size distribution was around 172 nm with ±8.39 for standard deviation.

### 3.5. Performance of Current CNFs Grown on RFSFAC Surface in Comparison to Other CNFs Produced from Biomass-Activated Carbon

Carbon nanofibers built from biomass have a wide range of qualities and features. Table 2 gives information about the summarization of biomass CNFs’ expected results from this study and some of the findings from previous studies.

## 4. Discussion

Generally, the characteristics of CNFs are determined by the growth conditions. D. Vincent et al. [4] states that there are four steps in the CNF growth mechanism, including the formation of catalyst nanoparticles, carbon adsorption at the catalyst site, carbon through a large portion, and/or diffusion of the catalyst surface, and nucleation. As a result, CNFs involve three main components: gases, an underlayer, and a catalyst. It is well known that the transition metals Fe, Ni, and Co have been used as a catalyst for CNF synthesis and significantly impact the morphology and performance of CNFs [55]. It is also known that biomass materials as activated carbon precursors can act as catalysts in the synthesis of carbon nanomaterials since some mineral elements are widespread in biomass matrices as essential trace elements for plant growth. Chen et al. [45] discovered CNF clumps on the surface of ACs created from Fe-containing shells of palm kernel, coconut, and wheat straw with the contents of iron in the activated carbon corresponding to 2.91%, 0.17%, and 1.79%, respectively. Their analysis results concluded that Fe particles derived from AC precursors could perform a catalytic role in the formation of CNFs. In addition, to element Fe, due to the significant mineral components of biomass, the functions of other elements during CNF/CNT production have attracted the interest of many researchers. For example, K. Shi et al. [56] reported that mineral matter in gumwood containing Si and Ca acts as a catalyst for the formation of CNTs during a microwave-induced pyrolysis process. The pyrolysis temperature was set to 500 °C and kept at that temperature for 30 min. The authors deduced that Si and Ca decompose volatiles produced by microwave-induced pyrolysis of gumwood, resulting in the formation of CNTs, where Si and Ca content elements were 0.02 wt% and 0.06 wt%, respectively. This mechanism of growth of CNFs/CNTs is known as the vapor–solid phase growth model [57]. It has also been reported by Zhang et al. [23] that 0.37% K in pine nut shells is also thought to partially play a catalytic role during the formation and growth of hollow CNFs because It can vaporize as a volatile content and, thus, has a high migration ability at high temperatures.

As discussed above, it shows that the element contents of biomass are generally to be a catalytic component during the preparation of CNFs/CNTs. As shown in Table 1, the CNFs on the surface of the RFSAC consisted of a high element of C, O, and P, but less than 1 % of Si and K. This demonstrated that no metal elements were found in CNFs based on RFSAC, implying that no metal elements played a catalytic role in the CNF growth. In this study, it can be assumed that even in the absence of a catalyst, the formation of CNFs on the surface of the RFSAC was due to the chemical composition contained in the RFS itself. According to the references, carbon can be dissolved in silicates to generate CNFs/CNTs [56,58]. During the pyrolysis process, elements in the RFS charcoal melted initially and formed droplets on the surface of the charcoal. The melt not only contained elements such as O, P, and K, but there might also have been silicate in it. As a result, it is logical to believe that Si and K may play an essential role in CNF formation during the process.

Another factor that could contribute to the production of CNFs in this study was the chemical composition of RFS. The RFS is a lignocellulosic substance that contains 48.64 percent cellulose [59] and between 35 and 54 percent lignin [6]. The RFS can be easily altered into nano-sized fiber due to the cellulose-linked and hydrogen bond [3]. Additionally, according to Pandia et al. [6], lignin is the most effective natural polymer for creating carbon nanofibers, particularly when phosphoric acid (H_3_PO_4_) is added to the lignin solution which results in more curled fibers. This result can be explained by the fact that, aside from the fact that the elemental composition of biomass (O, P, K, and Si) is generally considered to be a catalytic component during the preparation of CNFs/CNTs, but also by the fact that the RFS is a natural polymer that can be easily transported into CNFs. Thus, the use of rubber fruit shells as a raw material for CNF production is extremely promising, as it is abundant, renewable, and environmentally beneficial. Additionally, by employing an uncomplicated and straightforward approach that does not require the use of catalysts, does not require vacuum or gas, and is carried out at moderate temperatures, the manufacturing is reasonably safe/harmless and inexpensive.

## 5. Conclusions

CNFs were formed on the surface of the RFSAC (prepared with H_3_PO_4_ activation solution), continuing with the hydrothermal process at a low temperature of 90 °C, where vacuum and gas were not required and without the use of additional catalysts. A SEM and TEM analysis showed many great CNFs with varying diameters on the surface of the RFSAC, where the average diameter size distribution was around 172 nm. The results of the Raman spectroscopy analysis revealed an intensity maxima at around 1340 and 1582 cm^−1^ as D and G bands were equivalent to those of graphite for CNF characteristics. Similar results were obtained by the XRD analysis, where an amorphous graphitic carbon structure was formed. TGA and BET results revealed that CNFs have a high thermal stability and surface area 63 m^2^g^−1^ with an adsorption average pore size of 2.11 nm. Hence, based on the results demonstrated in this study, the RFS is considered an alternative renewable biomass carbon source for CNF preparation, with converting biomass into amorphous carbon through chemical activation and the hydrothermal process.

## Figures and Tables

**Table 1:** Elemental content quantitative analysis.

Element	Line	Intensity Corrn.	Atomic%
C	K_SERIES	0.6678	62.48
O	K_SERIES	0.5744	32.83
Si	K_SERIES	0.9733	0.22
P	K_SERIES	1.3567	4.38
K	K_SERIES	1.0071	0.09
Total			100

**Table 2:** The performance of carbon nanofiber prepared from various biomass raw materials.

Biomass	Synthesis Method	Catalyst	BET (m^2^/g)	CNF Diameter (nm)	Intensity Ratio (I_D_/I_G_)	Ref.
Bamboo (cellulose fibers)	Pyrolysis	-	na	10–30	0.84	[16]
Sawdust	Pyrolysis	Fe	360–421	na	0.9–1.4	[52]
Natural fungus	Hydrothermal technique at 150 °C	-	895–1280	620	1.4–1.55	[19]
Cellulose	Ultrasonication		865	200	1.21	[53]
Poplar lignin powders	Electrospinning		221–837	80–370	0.73–0.87	[54]
RFSAC	Hydrothermal low temperature at 90 °C	-	63	38–554 (172 average)	0.98	This work

Note: na, not available.

## Data Availability

Not applicable.
